# Supplementary material for: Multi-Analytical Approach to Characterize the Degradation of Different Types of Microplastics: Identification and Quantification of Released Organic Compounds
Source: Molecules. 2023 Feb 1;28(3):1382. doi: 10.3390/molecules28031382 (PMC9919824; doi:10.3390/molecules28031382)
Supplement: Supplementary file 1 [file molecules-28-01382-s001.zip › molecules-2164445-supplementary.pdf]

Table S1. Compounds found at t_0 and after 4 weeks (t_0+4w) of aging of the investigated polymers. HDPE: high density polyethylene; LDPE: low density polyethylene; PET: polyethylene terephthalate; PP: polypropylene; PS: polystyrene.

**Compounds**	**Tr (min)**	**HDPE at t_0**	**HDPE at t_0+4w**	**LDPE at t_0**	**LDPE at t_0+4w**	**PET at t_0**	**PET at t_0+4w**	**PP at t_0**	**PP at t_0+4w**	**PS at t_0**	**PS at t_0+4w**
Acetone	9,52	X	X	X	X	X	X	X	X		X
Acetic acid, methyl ester	11,49										X
Silanol, trimethyl-	14,23					X	X	X	X	X	X
Pentane, 2-methyl-	14,27							X			
Methacrolein	14,48					X					
Formic acid, 1-methylethyl ester	14,99										
Acetic acid	15,22	X	X	X	X	X	X	X		X	
Pentane, 3-methyl-	15,49							X			
Methyl vinyl ketone	15,79		X		X				X	X	
2,3-Butanedione	16,06										
Butanal	16,32	X		X	X		X				
Propanal, 2-methyl-	16,35							X			
n-Hexane	16,78							X			X
2-Butanone	16,79			X			X		X		X
Furan, 3-methyl-	17,07					X					
Trichloromethane	18,94										
Tetrahydrofuran	19,35	X		X		X	X			X	
Ethyl Acetate	19,36			X					X	X	X
Cyclopentane, methyl-	19,42							X			
Cyclopentene, 3-methyl-	19,91								X		
2-Propanone, 1-hydroxy-	21,79										
Benzene, pentafluoro-	21,92			X	X	X	X	X	X	X	X
Cyclohexane	22,15								X		
Benzene	22,46	X	X	X				X			X
1-Propen-2-ol, acetate	22,54								X		
Furan, 2,3-dihydro-5-methyl-	22,91	X									
Propanoic acid	23,17	X					X			X	
2-Propenoic acid	23,26						X				
2-Pentanone	23,53					X	X		X	X	X
2-Butanone, 3-methyl-	23,55						X				

Pentane, 2,2,4-trimethyl-	23,76										
Hexane, 2,2-dimethyl-	23,76					X					
Isopropylcyclobutane	23,86										
3-Pentanone	24,19								X		X
Pentanal	24,34										
Allyl acetate	24,34										
Pentane, 2,2,4-trimethyl-	24,36		X								
Methane, oxybis[chloro-	24,38										
Pentanal	24,47		X	X	X						X
(R)-(-)-2-Pentanol	24,47								X		
Heptane	24,48					X	X				
2-Propenoic acid	24,55								X		
Furan, 2,5-dimethyl-	24,90								X		
n-Propyl acetate	25,33								X		
2,4-Dimethylfuran	25,46					X			X		
Furan, 2-methoxy-	25,52								X		
Furan, 2,5-dihydro-3,4-dimethyl-	26,19										
2-Pentanol, 2-methyl-	26,79								X		
2-Butanol, 2,3-dimethyl-	26,80										
3-Penten-2-one, (E)-	27,02								X		
3-Penten-2-one	27,05									X	
Methyl Isobutyl Ketone	27,13								X		
1-Methylcyclohexa-1,3-diene	27,42								X		
Pyridine	27,76					X				X	
1,6-Heptadiene, 2-methyl-	27,76										
Heptane	27,82	X									
sec-Butyl acetate	28,20								X		
2-Heptene, 4-methyl-, (E)-	28,31										
Acetic anhydride	28,89										
Heptane, 4-methyl-	28,92							X			
Toluene	29,19						X	X		X	X
Pyridine	29,19	X									
Spiro[2,4]hepta-4,6-diene	29,20	X	X								

Heptane, 3-methyl-	29,32										
Butanoic acid	29,50								X		
Acetylacetone	29,76								X		
2-Butenoic acid, (E)-	29,84								X		
CH3C(O)CH2CH2OH	30,07								X		
2-Hexanone	30,17										X
Toluene	30,18			X							
Pentanal, 2-methyl-	30,18										
Butane, 1-(2-propenyloxy)-	30,18										
2-Pentanone, 3-methylene-	30,29										
1-Methylallyl acetate	30,41										
Cyclopentanone	30,55										X
1,3-Cyclohexadiene, 5,6-dimethyl-	30,57										
2-Butenal, 2-methyl-	30,57										
Propanoic acid, TMS derivative	30,60								X		
3-Penten-2-one, 4-methyl-	30,77										
Furan, 2-methoxy-	30,77						X			X	X
3-Penten-2-one, 4-methyl-	30,77					X					
1,1-Ethanediol, diacetate	30,90								X		
2-Hexanone	30,93				X						
Octane	30,93							X			
Hexanal	30,93					X	X			X	X
2-Butenoic acid, (E)-	31,06						X				
Acetic acid, butyl ester	31,56					X	X				X
Furan, 2,3,5-trimethyl-	31,75								X		
2-Pentanone, 4-hydroxy-	31,94					X			X		
Heptane, 2,4-dimethyl-	32,20							X			
Heptane, 3-methyl-	32,20										
Ethanol, 2-(1,1-dimethylethoxy)-	32,45							X			
Benzonitrile, pentafluoro-	32,68						X				
2-Pentanone, 4-hydroxy-4-methyl-	33,27					X			X		

2,4-Dimethyl-1-heptene	33,33							X			
2-Pentanol, acetate	33,45								X		
1,1-Ethanediol, diacetate	33,45								X		
3-Oxetanol, 2,2,3-trimethyl-	33,60										
1,3-Cyclohexadiene, 5,6-dimethyl-	33,68										
Cyclopentadiene, 2,5,5-trimethyl-	33,68								X		
2-Hexanone, 5-methyl-	34,12										
Butanoic acid, 3-hydroxy-, methyl ester, (S)-	34,13								X		
2-Propanone, 1-(acetyloxy)-	34,31					X	X		X	X	X
m-Dioxane, 4,5,5-trimethyl-	34,78								X		
Unk_1	34,80	X	X	X	X						
Ethylbenzene	34,81							X			
2(3H)-Furanone, 5-methyl-	34,82								X		
1,2-Propanediol, 1-acetate	35,11										
Ethylbenzene	35,26	X								X	X
Benzene, 1,3-dimethyl-	35,27					X		X		X	
4-Cyclopentene-1,3-dione	35,53										X
Hexan-2,4-dione, enol	35,55								X		
3-Heptanone	35,68										
1,2-Propanediol, 2-acetate	35,70								X		
2-Pentanone, 4-hydroxy-4-methyl-	35,80					X			X		
2-Heptanone	35,87										X
Benzene, 1,3-dimethyl-	35,87	X	X	X							
2-METHYLPENTYL FORMATE	35,90										
Cyclobutane, butyl-	36,02										
2-Heptanone	36,03										
1-Pentanol, 4-methyl-	36,03										
1-Nonene	36,03										
4-Methyl-2-pentyl acetate	36,04										
1-Hexanol	36,12										

Ethyl orthoformate	36,20										
3-Heptanol	36,26										
Nonane	36,48										
Styrene	36,49					X		X		X	X
Pentafluorobenzaldehyde	36,49	X	X								
Ethylbenzene	36,58							X			
p-Xylene	36,58								X		
Benzene, 1,3-dimethyl-	36,58										
Heptanal	36,58										
3,3-Dimethyl-2,4-pentane dione	36,98								X		
2(5H)-Furanone	37,01				X		X				X
Styrene	37,15	X									
Butanoic acid, 4-hydroxy-	37,16				X				X		X
1,2-Propanediol, 2-acetate	37,44								X		
Acetic acid, diethyl-	37,56										
2,5-Hexanedione	37,74			X	X	X	X	X		X	X
Butanoic acid, 4-hydroxy-	37,74				X						
Cyclopentanone, 3-methyl-	37,83										X
Pentanoic acid, 3-methyl-	37,96										
Benzene, (1-methylethyl)-	38,19									X	X
Butanoic acid, 3-hydroxy-, ethyl ester	38,23										
2-Heptanone, 4-methyl-	38,43								X		
Benzene, (chloromethyl)pentafluoro-	38,68			X	X	X	X	X	X	X	X
2,5-Hexanedione	38,69										
Bicyclo[3.1.0]hex-2-ene, 2-methyl-5-(1-methylethyl)-	38,97						X	X			
2-Butyn-1-al diethyl acetal	39,30										
Benzene, (chloromethyl)pentafluoro-	39,42	X	X								
Octane, 4-ethyl-	39,46	X									
2-Acetyl-2-methyltetrahydrofuran	39,46				X						
Pentanoic acid	39,53								X		

2(3H)-Furanone, dihydro-5-methyl-	39,57		X		X				X		
Pentanoic acid	39,84				X						
Heptane, 2,2,3,5-tetramethyl-	39,92										
Octane, 2,2,6-trimethyl-	39,92										
2H-Pyran-2-one, tetrahydro-	39,94										
Hexanoic acid	39,94				X						
1-Heptanol	40,18										
2,4-Pentanedione, 3-ethyl-	40,21								X		
2-Cyclopenten-1-one, 3-methyl-	40,36										
3-Isopropyl-5-methylhexan-2-one	40,46										
Phenol	40,47							X			X
Benzaldehyde	40,54		X	X	X	X	X	X		X	X
2-Butanone, 4-(acetyloxy)-	40,66					X			X		
Octane, 2,2,6-trimethyl-	40,74										
Benzene, 1,2,4-trimethyl-	40,76								X		
Pentanoic acid, 4-oxo-, methyl ester	40,95										X
1,9-Decadiene	41,02										
1H-Pyrrole-2,5-dione	41,10					X		X			
3-Octanone	41,11										
3-Pentenoic acid, 4-methyl-	41,14										X
Benzaldehyde	41,31	X	X								
2-Octanone	41,32										X
Cyclopropane, 1,2-dimethyl-1-pentyl-	41,41										
1-Decene	41,42										
Furan, 2-pentyl-	41,50		X	X	X		X				X
tert-Butylamine, N-ethyl-N-nitroso-	41,55							X			
Heptane, 2,2,4,6,6-pentamethyl-	41,69	X									
Decane	41,80					X		X		X	
Octane, 2,7-dimethyl-	41,81			X							
2-Furanone, 2,5-dihydro-3,5-dimethyl	41,85								X		

Octane, 2,6,6-trimethyl-	41,98										
Octanal	42,01			X	X	X	X	X		X	X
Decane	42,01	X									
Nonane, 2,6-dimethyl-	42,17							X			
2(5H)-Furanone, 3,5,5-trimethyl-	42,24								X		
1,2-Propanediol, diacetate	42,31								X		
Nonane, 2,6-dimethyl-	42,35							X			
Benzofuran	42,37										X
Heptane, 2,2,4,6,6-pentamethyl-	42,56				X						
Propanoic acid, 2-methyl-, anhydride	42,69										
Benzene, (2-methylpropyl)-	42,70									X	
Benzene, (bromomethyl)pentafluoro-	42,81	X	X	X	X	X	X	X	X	X	X
Octanal	42,81	X	X								
Heptane, 5-ethyl-2,2,3-trimethyl-	42,82										
Formic acid, heptyl ester	42,94										
Acetic acid, 1-methyl-3-oxo-but-1-enyl ester	42,98								X		
Tetrasiloxane, decamethyl-	43,23										
Heptane, 2,2,4,6,6-pentamethyl-	43,23										
Acetic acid, (acetyloxy)-	43,30										
2,2,4,4-Tetramethyloctane	43,39										
Formic acid, heptyl ester	43,54										
Octane, 6-ethyl-2-methyl-	43,55				X						
Benzyl alcohol	43,56										X
Octane, 2,6,6-trimethyl-	43,57	X									
6-Heptenoic acid	43,86				X						
Propanoic acid, 2-methyl-, anhydride	43,88										
Hexanoic acid	43,99					X	X				
Nonane, 4,5-dimethyl-	44,08							X			
Benzeneacetaldehyde	44,09						X			X	X
Heptanoic acid	44,15				X						

N-Dimethylaminomethyl-tert.-butyl-isopropylphosphine	44,18										
2(3H)-Furanone, 5-ethyldihydro-	44,24										X
Benzyl alcohol	44,24	X									
Decane, 2,6,7-trimethyl-	44,27			X							
Heptane, 5-ethyl-2,2,3-trimethyl-	44,27										
Benzaldehyde, 2-hydroxy-	44,31										X
Acetic acid, phenyl ester	44,36										X
Heptane, 4-ethyl-2,2,6,6-tetramethyl-	44,48										
Benzenemethanol, .alpha.-methyl-	44,59										X
5-Nonanone	44,69										
2(3H)-Furanone, 5-ethyldihydro-	44,95										
Acetophenone	44,95	X	X	X	X	X	X	X	X	X	X
7-Azabicyclo[4,2,0]octan-8-one	45,04										
Pentanoic acid, 2-methyl-4-oxo-	45,07										
Formic acid, phenylmethyl ester	45,16										X
2-Butenoic acid, butyl ester	45,22										
2-Nonanone, 3-(hydroxymethyl)-	45,28										
Cyclopropane, octyl-	45,30				X						
5-Hydroxy-4,5-dimethyl-2,5-dihydrofuran-2-one	45,34					X	X		X		
2-Nonanone	45,56										
Decane, 2,4-dimethyl-	45,56										
Undecane	45,56	X						X		X	
1,5-Dimethyl-2-pyrrolecarbonitrile	45,62										
2(3H)-Furanone, 5-ethyldihydro-5-methyl-	45,64										
2H-Pyran-2-one, tetrahydro-6-methyl-	45,70				X						
Benzoic acid, methyl ester	45,82										X
Benzeneacetyl chloride, 2,3,4,5,6-pentafluoro-	46,36			X							
Formic acid, octyl ester	46,39										

Formic acid, 1-phenylethyl ester	46,45										X
Nonanal	46,79	X	X	X	X	X	X	X		X	X
Benzyl methyl ketone	46,79										X
1-Undecene, 9-methyl-	46,96										
Cyclohexane, pentyl-	47,06										
5-Ethyldecane	47,35	X									
Ethanone, 1-(6-methyl-7-oxabicyclo[4.1.0]hept-1-yl)-	47,40								X		
2(3H)-Furanone, dihydro-5-propyl-	47,47		X								X
Decane, 5-methyl-6-methylene-	47,56	X									
Acetic acid, phenylmethyl ester	47,56										X
5-Decanone	47,61										
Undecane, 3-methyl-	47,62	X									
1-Propanone, 1-phenyl-	47,86										X
1,11-Dodecadiene	47,89										
4-Decanone	47,89										
Ethanone, 1-(2-hydroxyphenyl)-	47,90										X
3-Decanone	48,08										
1-Dodecene	48,10	X									
2-Decanone	48,36										
Benzene, 1,3,5-trichloro-	48,48			X							
3-Dodecene, (Z)-	48,49	X									
Decanal	48,62	X	X	X	X	X	X	X		X	X
Methyl salicylate	48,65										X
Undecane, 2-methyl-	48,69										
3-Dodecene, (E)-	48,70	X									
Naphthalene	48,86							X			
2,7-Nonadien-5-one, 4,6-dimethyl-	48,92										
1-Nonanol	48,92				X						
Ethanol, 2-phenoxy-	49,16	X									
5-Methyl-2-(2-methyl-2-tetrahydrofuryl)tetrahydrofuran	49,32										

Butanoic acid, 3-hexenyl ester, (Z)-	49,51										
Cyclobutanecarboxylic acid, 3-methylbut-2-enyl ester	49,55	X									
Undecane, 5-methyl-	49,67	X									
Benzothiazole	49,75									X	
Benzene, 1,3-bis(1,1-dimethylethyl)-	49,76		X								
1,2-Benzisothiazole	49,76	X						X			
Benzothiazole	49,79		X								
1-Methoxydecane	49,84										
2(3H)-Furanone, dihydro-5-propyl-	49,93		X								
Undecane, 3-methyl-	50,09	X									
1,11-Dodecadiene	50,09										
Octyl chloroformate	50,23					X					
1-Nonanol	50,23										
1-Dodecene	50,23	X									
Undecane, 4,7-dimethyl-	50,37					X					
Oxalic acid, butyl propyl ester	50,39										
Cinnamaldehyde, (E)-	50,40										X
Cyclodecane, methyl-	50,49	X									
Decanal	50,62					X	X				
Undecane, 2,3-dimethyl-	50,64	X									
Undecane, 4-ethyl-	50,64	X									
Undecane, 6-ethyl-	50,94	X									
Dodecane, 2,6,11-trimethyl-	50,98							X			
Dodecane, 2,6,11-trimethyl-	50,98										
Undecane, 3,8-dimethyl-	51,14							X			
Undecane, 4,7-dimethyl-	51,14										
1-Decanol	51,16		X								
Dodecane, 5-methyl-	51,32					X					
2-Undecanone	52,91										
Dodecane, 4,6-dimethyl-	52,97							X			

1-Tridecene	53,69										
Tridecane, 5-methyl-	54,58										
n-Nonylcyclohexane	55,21				X						
Benzene, (1-butylheptyl)-	56,53										
Dodecane, 1-iodo-	56,60										
Hexadecane	56,61			X	X						
Diphenyl sulfide	56,72							X			X
2-Decanone	56,72										
Cyclopropanecarboxamide, N-(3-methylphenyl)-	57,62						X				
Benzoic acid, phenyl ester	57,91										
